# P-1287. In Vitro Activity of Cefiderocol against Resistant Subsets of Pseudomonas aeruginosa collected from United States Hospitals during 2020–2024

**DOI:** 10.1093/ofid/ofaf695.1475

**Published:** 2026-01-11

**Authors:** Rodrigo E Mendes, Joshua Maher, Zachary Kockler, John Kimbrough, Mariana Castanheira

**Affiliations:** Element Iowa City (JMI Laboratories), North Liberty, IA; Element Iowa City (JMI Laboratories), North Liberty, IA; Element Iowa City (JMI Laboratories), North Liberty, IA; Element Iowa City (JMI Laboratories), North Liberty, IA; Element, North Liberty, IA

## Abstract

**Background:**

*Pseudomonas aeruginosa* (PSA) has intrinsic treatment-limiting resistance mechanisms, which decrease antibiotic permeability, and has also the ability to acquire resistance. Cefiderocol (FDC) is a siderophore cephalosporin that uses the iron transport systems of Gram-negative bacteria to enhance cell entry. The activity of FDC and comparators was evaluated against PSA causing infections in US hospitals, including resistant subsets, as part of the SENTRY Antimicrobial Surveillance Program.

**Figure:**
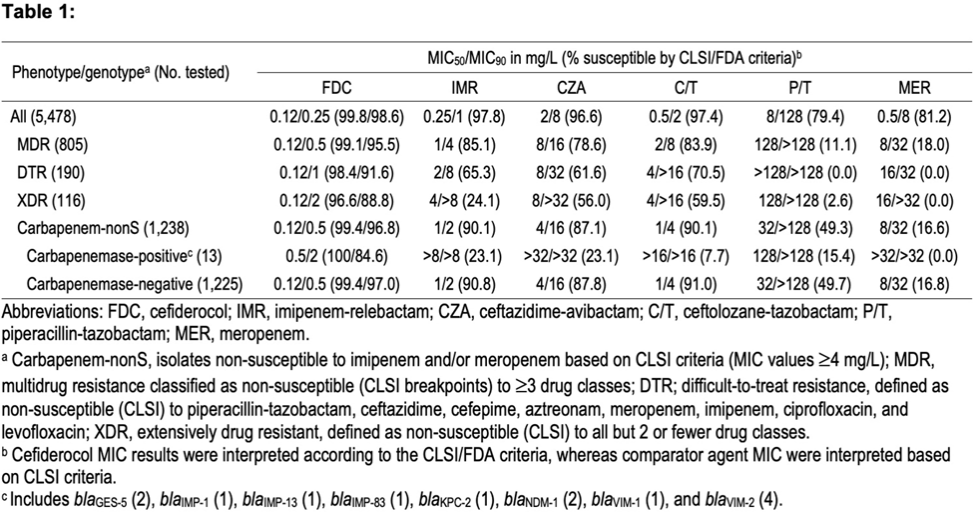

**Methods:**

5,478 PSA were collected from 38 sites in the US in 2020–2024. Susceptibility (S) testing was performed by broth microdilution with cation-adjusted Mueller-Hinton broth (CAMHB) for comparators and iron-depleted CAMHB for FDC. CLSI/FDA criteria were applied. Isolates with imipenem (IMI) or meropenem MIC ≥4 mg/L (nonS by CLSI) were screened for β-lactamase genes. Mutidrug resistance (MDR), difficult-to-treat resistance (DTR), and extensively drug resistance (XDR) are described in the footnotes of Table 1.

**Results:**

14.7% (805/5,478), 3.5% (190/5,478) and 2.1% (116/5,478) PSA isolates were MDR, DTR and XDR, respectively. 22.6% (1,238/5,478) PSA were carbapenem-nonS, with only 13 (1.1%) carrying carbapenemases (Table 1). FDC and β-lactam/β-lactamase inhibitor (BL/BLI) combinations showed S of >95% against all PSA, except for piperacillin-tazobactam (79.4%S). FDC (88.8–99.1%S) had MIC_50_ of 0.12 mg/L and MIC_90_ of 0.5–2 mg/L against MDR, DTR, and XDR isolates, whereas BL/BLI showed S of ≤85.1%. FDC (96.8–99.4%S) had the lowest MIC against carbapenem-nonS PSA with an MIC_90_ ≥4-fold lower than comparators. FDC (100%S) inhibited all PSA carrying carbapenemases at the CLSI S breakpoint (≤4 mg/L), whereas BL/BLI combinations showed S of ≤23.1%. FDC (MIC_50/90_, 0.12/0.5 mg/L; 97.0–99.4%S), IMI-relebactam (MIC_50/90_, 1/2 mg/L; 90.8%S) and ceftolozane-tazobactam (MIC_50/90_, 1/4 mg/L; 91.0%S) were active ( > 90%S) against carbapenem-nonS PSA absent of carbapenemase genes.

**Conclusion:**

FDC showed potent activity against PSA isolates from the US. FDC was active against highly resistant subsets, where cross resistance among comparators was noted. These *in vitro* data suggest FDC as an important option for the treatment of infections caused by PSA.

**Disclosures:**

Rodrigo E. Mendes, PhD, GSK: Grant/Research Support|Shionogi & Co., Ltd.: Grant/Research Support|United States Food and Drug Administration: FDA Contract Number: 75F40123C00140 Mariana Castanheira, PhD, Melinta Therapeutics: Advisor/Consultant|Melinta Therapeutics: Grant/Research Support

